# Three-Dimensional Printing Model-Guided Percutaneous Closure of Atrial Septal Defect

**DOI:** 10.5935/abc.20170051

**Published:** 2017-05

**Authors:** Hongxing Luo, Yu Xu, Zhongmin Wang, Yuhao Liu, Chuanyu Gao

**Affiliations:** Zhengzhou University People's Hospital, Zhengzhou, Henan Province - China

**Keywords:** Heart Septal Defects, Atrial / surgery, Echocardiography / methods, Imaging, Three-Dimensional

A 32-year-old female with a 2-year history of chest distress was admitted to our department due to exacerbation for 3 days. On physical examination, we found fixed splitting second heart sounds on the patient's pulmonic area. An echocardiography was performed and showed a 15-mm atrial septal defect of the inferior vena cava type. After obtaining the patient's consent, a three-dimensional printing cardiac model was printed out. We tried various sizes of ASD occluders on the model to completely cover the defect, which indicated that a 28-mm occluder was appropriate. Thus, we placed a 28-mm ASD occluder during the operation and succeeded after only one attempt. The patient was re-assessed by echocardiography, which showed a favorable position of the ASD occluder without any left-to-right shunt.

Three-dimensional printing (3D printing) is a new technology that converts two-dimensional medical images into a tangible object, allowing not only a comprehensive view of the cardiac anatomical structures but also preoperative simulation to choose the optimal size of ASD occluder. Although it has been applied to orthopedics, general surgery and so on, the use of 3D printing in cardiology is still at its infancy. Our case showed the feasibility of using a 3D printing cardiac model to guide the percutaneous closure of ASD. It is likely to increase the success rate and reduce the operation time for interventional cardiology, especially the complex ASD cases, and more studies should be carried out to extend its fields of application.

**Figure 1:**
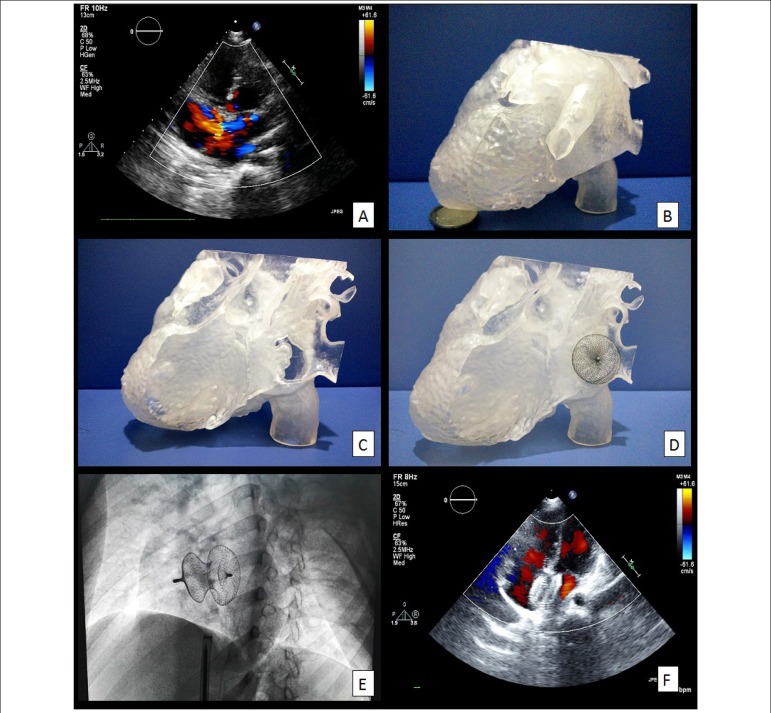
Echocardiographic apical four-chamber axis view showing a 15-mm ASD with left-to-right shunt (A). Three-dimensional printing cardiac model in whole view (B) or being separated to show the ASD (C). A 28-mm occluder was placed to completely cover the ASD (D). Intraoperative placement of a 28-mm ASD occluder after one trial (E). Postoperative echocardiographic apical four-chamber axis view showing no left-to-right shunt (F).

